# Informational Content of the VIX Index: Dynamic Entropy Approach

**DOI:** 10.3390/e28050528

**Published:** 2026-05-06

**Authors:** Joanna Olbryś, Dawid Toczydłowski

**Affiliations:** Faculty of Computer Science, Bialystok University of Technology, Wiejska 45a, 15-351 Białystok, Poland; dawid.toczydlowski.113855@student.pb.edu.pl

**Keywords:** VIX Index, STSA, discretization, information theory, Shannon entropy, rolling-window

## Abstract

The aim of this study is to thoroughly assess the informational content of the CBOE Volatility Index^®^ (VIX^®^ Index) in the context of various turbulent periods. The VIX Index is especially important from an investor perspective. It is often referred to as the “investor fear gauge”, because its level tends to spike during periods of market turmoil and other extreme events. Therefore, this index significantly differs from other market indices and financial instruments. Information theory and normalized Shannon entropy, combined with a rolling-window dynamic approach, are used to explore the evolution of the VIX Index over time. The research hypothesis states that the informational content of the VIX Index varies substantially across periods affected by crucial events. To verify this hypothesis, three important periods of the twenty-first century are analyzed: (1) the Global Financial Crisis, (2) the COVID-19 pandemic outbreak, and (3) the period covering the sub-periods before and after the Donald Trump’s Presidential Inauguration. The results provide no reason to reject the research hypothesis. The empirical findings show that the entropy values appear to be quite sensitive to the choice of discretizaton procedure. However, this evidence is consistent with the existing literature.

## 1. Introduction

The CBOE Volatility Index^®^ (VIX^®^ Index) is a measure of market expectations of near-term volatility derived from S&P 500 Index^®^ (SPX) option prices. Since its introduction in 1993 [1], the VIX^®^ Index has been regarded as the world’s leading barometer of investor sentiment and market volatility [2]. Whaley [3] points out that the VIX Index is called the “investor fear gauge”, and this description is appropriate because the VIX level tends to spike during periods of market turmoil and various extreme events (e.g., substantial stock market declines, threats of war, or unexpected changes in interest rates). The index reflects investors’ consensus view of expected future stock market volatility. In general, the higher the VIX, the higher the level of fear. The descriptor “fear” arises from the fact that investors are risk averse [3]. Further details concerning the VIX calculation are described in Section 2.

The VIX Index provides a reliable estimate of expected short-term market volatility which is a critical input for many investment decisions [1]. It should be viewed as an important source of market information for investors. Therefore, the informational content of the VIX Index and the degree of regularity or irregularity in its time series are particularly interesting research issues.

The informational content of financial time series can be investigated using symbolic time series analysis (STSA) and entropy-based procedures. In the light of the existing literature, STSA offers several important advantages in finance. These procedures make it possible to capture dynamic time-varying patterns in successive values of financial time series. Moreover, data symbolization converts a discrete data series with many possible values into a symbolic series containing only a few distinct values. Various discretization methods can effectively filter the data and reduce noise. Importantly, symbolic encoding of information forms the foundation of Shannon’s mathematical theory of communication and the seminal concept of information entropy. In this study, normalized Shannon entropy based on a symbolic encoding procedure with two thresholds [4] is used to explore the informational content of changes in the VIX Index. Furthermore, alternative ternary encoding procedures are utilized to perform robustness analyses.

The contribution of this study is twofold. First, our paper directly investigates the informational content of the VIX Index using information theory and the Shannon entropy. In addition, a rolling-window dynamic approach is employed to explore the evolution of normalized Shannon entropy over time. In the existing literature, there are several papers that investigate relationships and information flow between the VIX Index and other financial time series using entropy-based methods (see, e.g., [5,6]), but these papers do not assess the informational content of the VIX. One paper [7] investigates the VIX response to the COVID-19 pandemic crisis using different entropy-based methods, but the analyzed time period is different (1995–2020).

Second, the research hypothesis states that the informational content of the VIX Index varies substantially across periods affected by crucial events. To verify this hypothesis, three important periods of the twenty-first century are analyzed: (1) the Global Financial Crisis, (2) the COVID-19 pandemic outbreak, and (3) the period covering the sub-periods before and after the Donald Trump’s Presidential Inauguration. The dataset is relatively long, as it covers the period from the beginning of 2006 to the end of 2025. Importantly, all major findings are illustrated with symbol-sequence histograms and other relevant charts. The results provide no reason to reject the research hypothesis. However, the findings show that the entropy values appear to be quite sensitive to the choice of coding procedure, which is consistent with the previous studies [4,8,9]. The empirical results reported in this paper are novel and, to the best of the authors’ knowledge, have not been presented in the literature thus far.

The paper is organized as follows. Section 2 presents some basic information about the VIX Index. Section 3 describes the methodological background. Section 4 contains the results of empirical experiments concerning the assessment of informational content of the VIX time series within the long period from January 2006 to December 2025 (twenty years). Finally, Section 5 outlines the main conclusions.

## 2. The CBOE Market Volatility Index (VIX)

In this short section, the methodological background of the CBOE Market Volatility Index (VIX) calculation is presented in detail. The VIX Index is a financial benchmark that measures the level of expected volatility of the S&P 500 Index over the next thirty days that is implied in the bid/ask quotations of SPX options. Therefore, the VIX Index is a forward-looking measure, in contrast to realized (or actual) volatility, which measures the variability of historical (or known) prices [2]. Based on [2], the generalized formula used in the VIX Index calculation is given by the following Equation (1):

(1) $$\sigma^{2}=\frac{2}{T}\sum_{i}\frac{\Delta K_{i}}{K_{i}^{2}}\exp\left(RT\right)Q\left(K_{i}\right)-\frac{1}{T}\left[\frac{F}{K_{0}}-1\right],$$

where

*T*—time to expiration,
*F*—forward index level derived from index option prices,
*R*—risk-free interest rate to expiration,
$K_{0}$—first strike below the forward index level *F*,
$K_{i}$—strike price of *i*-th out-of-the-money option; a call if $K_{i}>K_{0}$ and a put if $K_{i}<K_{0}$; both put and call if $K_{i}=K_{0}$,
$Q(K_{i})$—the midpoint of the bid-ask spread for each option with strike $K_{i}$,
$\Delta K_{i}$—interval between strike prices; it is equal to half the difference between the strike on either side of $K_{i}$: $\Delta K_{i}=\frac{K_{i+1}-K_{i-1}}{2}$.
  (2) VIX=σ·100,
  where $\sigma^{2}$ is given by Equation (1).

As mentioned in the Introduction, the VIX Index was introduced to provide a benchmark of expected short-term market volatility [1]. This is a forward-looking index, and it measures the volatility expected by investors. It represents a market consensus forecast of stock market volatility over the next thirty calendar days. In [10], one can find further detailed discussion concerning the VIX Index estimation and interpretation.

There are several theoretical and practical applications of the VIX Index. Based on the literature, a broad research field concerns the variance premium and stock market volatility. In this context, the Global Financial Crisis (GFC) has intensified the need for indicators of the risk aversion of market participants. For instance, the behavioral finance literature has developed the so-called sentiment indices, and financial institutions have created a wide variety of risk aversion indicators. An increase in risk aversion should lead to a rise in risk premia across all markets, but the rise should be higher on the riskiest markets [11]. Among others, the implied volatility of options can be used to provide an indication of the amounts investors are prepared to pay to protect themselves from the risk of price fluctuations. As emphasized in the paper [12], since the VIX can be treated as the risk-neutral expected stock market variance for the S&P500 index, it could be utilized to calculate the equity variance premium. For a comprehensive review of the VIX Index premium, see [13] and the references therein.

## 3. Materials and Methods

This section presents the methodological background concerning the assessment of informational content of time series in the context of the Shannon’s [14] mathematical theory of information and communication. The ternary discretization procedure, symbol-sequence histograms, symbol-sequence statistics, and normalized Shannon entropy based on symbol-sequence histograms are described in detail.

### 3.1. The Ternary Discretization Including Volatility Estimates

In this study, the VIX Index time series is investigated. Daily logarithmic changes in the VIX level are computed as follows:

(3) $$r_{t}=lnP_{t}-lnP_{t-1},$$

where $P_{t}$ is the closing value of the VIX Index on day *t*.

The symbolization of the VIX Index changes time series $\left\{r_{t}\right\}$ into sequences of symbols $\left\{s_{t}\right\}$ and is performed with the use of the ternary encoding procedure with two thresholds, proposed in [4]. Empirical comparative experiments presented in [4] confirmed that the ternary discretization that includes volatility estimates is useful in assessing informational content of financial time series, since it enables investors to incorporate significant changes in time series values, specifically within various extreme event periods.

In the case of ternary discretization, the finite set A={0,1,2} of possible n = 3 symbols is called an alphabet, while each subset of a sequence of symbols is called a word. A sequence of consecutive time series values is symbolized as a sequence of 0 s, 1 s and 2 s. Analogical notation is proposed in several studies, for instance [8,14,15,16,17,18,19,20].

Based on [4], the encoding procedure is defined as follows:

**Definition** **1.** *A sequence $\left\{s_{t}\right\}$ of symbols is defined according to the following:*

(4) $$s_{t}=\left\{\begin{array}{cc} 0 & if\;r_{t}\leq \left(\bar{r}-\sigma \right)\\ 1 & if\;\left(\bar{r}-\sigma \right)<r_{t}\leq \left(\bar{r}+\sigma \right)\\ 2 & if\;r_{t}>\left(\bar{r}+\sigma \right) \end{array}\right.$$

*where $\bar{r}$ is the mean of logarithmic changes time series, σ is the standard deviation of logarithmic changes, and $\left(\bar{r}-\sigma \right)$ and $\left(\bar{r}+\sigma \right)$ are the thresholds of the time series.*

To perform robustness analyses, three additional ternary discretization procedures are used (see Appendix A). Definitions A1 and A2 are the modifications of Definition 1, while Definition A3 is the variant of the STSA method with the 5% and 95% sample quantiles as the thresholds [4,8,21,22].

In this study, the k = 3 length of a code sequence is used. Therefore, the number of all possible symbol sequences is equal to $n^{k}=3^{3}=27$. However, various code-sequence lengths (i.e., k = 3, k = 4, and k = 5) can be utilized, but the results presented in the literature confirm that the choice of a sequence length (k) is a minor issue, since the findings for different k values are very similar. Unfortunately, since the total number of sequences (equal to $n^{k}$) is large, visualization with histograms in the case of k = 4 or k = 5 is much more difficult [8].

### 3.2. Symbol-Sequence Histograms and Related Statistics

The structure of patterns in real data can be represented by the relative frequency of each possible *k*-length symbol sequence. The observed dynamics can be visualized by a *k* histogram of relative frequencies. The empirical distribution expressed by a histogram allows a researcher to compare coded sequences. On the basis of the literature, visualization of the frequencies with histograms makes it possible to conveniently observe patterns in time series (see, e.g., [8,15,16,23,24,25]).

The literature indicates that it is well-founded to assess a similarity between two histograms. Therefore, several measures of the distance between two histograms have been proposed and used in the literature in various applications (see, e.g., [26,27] and the references therein). Two of the most used distance metrics are (1) the Manhattan distance and (2) the Euclidean distance.

The Manhattan distance is defined by the following Equation (5):

(5) $$M_{XY}\left(k\right)=\sum_{i}\left|X_{i}-Y_{i}\right|,$$

where $X_{i}$ and $Y_{i}$ are the empirical frequencies of individual sequences for the sequence codes (*i*), given *k*-histograms *X* and *Y*.

The Euclidean distance is defined by Equation (6):

(6) $$E_{XY}\left(k\right)=\sqrt{\sum_{i}{\left(X_{i}-Y_{i}\right)}^{2}},$$

where the notation is the same as in Equation (5).

Both the Manhattan and Euclidean norms work like metrics in the space of all possible sequences (words), providing measures of the distance between different *k* histograms. Generally, longer distances indicate that the dynamics in the data are increasingly different.

### 3.3. Normalized Shannon Entropy Based on Symbolized Time Series

The Shannon entropy [14] of the *k*-th order (Definition 2) is an information indicator for symbol-sequence frequencies, and it quantifies the expected value of information contained in a discrete distribution.

**Definition** **2.** *The Shannon entropy of the k-th order (S(k)) is defined as follows:*

(7) $$S\left(k\right)=-\sum_{i}p_{i}\cdot log_{2}\left(p_{i}\right),$$

*where $p_{i}$ is the probability of finding the i-th sequence of length k.*

The probability $p_{i}$ is approximated by the number of times the *i*-th sequence is found in the original symbolic string divided by the number of all non-zero sequences of length *k*. In the papers [4,8,16,20,24,25], the following Definition 3 of the normalized form of the Shannon entropy is used.

**Definition** **3.** *Normalized Shannon entropy ($0\leq S_{n}\left(k\right)\leq 1$) based on the symbolic representation of time series is defined as follows:*

(8) $$S_{n}\left(k\right)=\frac{-1}{log_{2}N}\cdot \sum_{i}p_{i}\cdot log_{2}\left(p_{i}\right),$$

*where N is the total number of observed sequences of length k with non-zero frequency, i is the index of a sequence, and $p_{i}$ is the probability of finding the i-th sequence of length k. It is assumed that $0\cdot log_{2}0=0$.*

## 4. Experimental Results and Discussion

This section describes the database and presents our comparative findings for the VIX Index within three periods affected by crucial events.

### 4.1. Real Data Description

The dataset includes daily observations of the VIX Index. The whole sample covers a long period from January 2006 to December 2025 (twenty years).

The VIX Index data were obtained free of charge from the Federal Reserve Bank of St. Louis (FRED) database, CBOE Volatility Index: VIX, series VIXCLS (https://fred.stlouisfed.org/series/VIXCLS, accessed on 3 January 2026).

All calculations were performed using a dedicated program prepared in Python (Jupyter Notebook 7.3.2 environment; Python 3.13.5).

**Figure 1:**
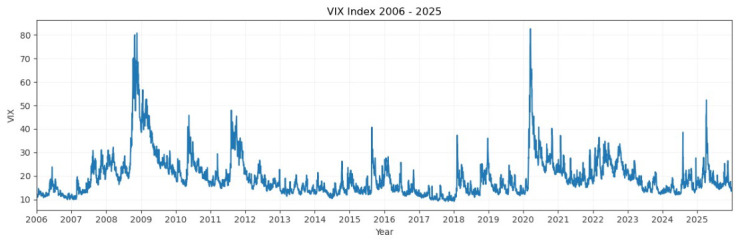
Daily VIX Index levels within the whole sample period (from January 2006 to December 2025).

Figure 1 illustrates the daily VIX Index levels during the whole sample period from January 2006 to December 2025. One can observe various significant jumps in the level of VIX, and this evidence is consistent with the literature. Over its history, the VIX Index has acted as a “fear index”, since high levels of VIX are coincident with high degrees of market turmoil [3].

As mentioned in the Introduction, in this research, three periods affected by crucial events are explored.

Chronologically, the Global Financial Crisis (GFC), as the first major financial crisis of the twenty-first century, is the first turbulent period investigated in our research. In the light of the existing literature, the 2007–2009 GFC originated in advanced economies, largely in the United States and the United Kingdom. The crisis transmission through financial channels was rapid. The GFC timeline in the U.S. was determined by the following four major events: (1) the increase in subprime delinquency rates in the spring of 2007, (2) the liquidity and credit crunch in late 2007, (3) the liquidation of Bear Stearns in March 2008, and (4) the bankruptcy of Lehman Brothers in September 2008 [28,29,30,31,32]. However, there is no unanimity in determining the phases of the GFC among the researchers. In this study, the period from October 2007 to February 2009 was assumed as the GFC period for the U.S. financial market (see, e.g., [28,32,33]).

The COVID-19 pandemic outbreak is the second investigated turbulent period. To determine the COVID-19 pandemic period, we use the World Health Organization (WHO) information [34]. It is assumed that this period started on 11 March 2020, since on this date the WHO officially declared the COVID-19 outbreak to be a global pandemic. By analogy, the COVID-19 period ended on 5 May 2023 based on the WHO declaration [35]. Two sub-samples of equal length are analyzed: the pre-COVID-19 period and the COVID-19 pandemic outbreak. The date 11 March 2020 is the middle critical point.

The third sub-sample comprises the period from 7 February 2024 to 31 December 2025 with the Donald Trump U.S. Presidential Inauguration date (20 January 2025) as the middle point. The date of the Presidential Inauguration is especially important, since at that point, Donald Trump legally assumed the role of president and started to make important economic and politically motivated decisions [25]. Undoubtedly, the U.S. presidential elections are crucial since they usually generate a high level of risk, not only in the United States but worldwide.

To assess the informational content of the VIX Index before and during the particular period, the following pairs of sub-periods of an equal length are investigated:

1. For the Global Financial Crisis (GFC):
  - The pre-GFC period from 2 May 2006 to 28 September 2007 (356 trading days);
  - The GFC period from 1 October 2007 to 27 February 2009 (356 trading days).
2. For the COVID-19 pandemic outbreak:
  - The pre-COVID-19 pandemic period from 3 January 2017 to 10 March 2020 (801 trading days);
  - The critical point: 11 March 2020;
  - The COVID-19 pandemic period from 12 March 2020 to 5 May 2023 (801 trading days).
3. For the U.S. 2025 Presidential Inauguration:
  - The period before the 2025 Donald Trump Presidential Inauguration from 7 February 2024 to 17 January 2025 (245 trading days);
  - The critical point: 20 January 2025;
  - The period after the 2025 Donald Trump Presidential Inauguration from 21 January 2025 to 31 December 2025 (245 trading days).

**Figure 2:**
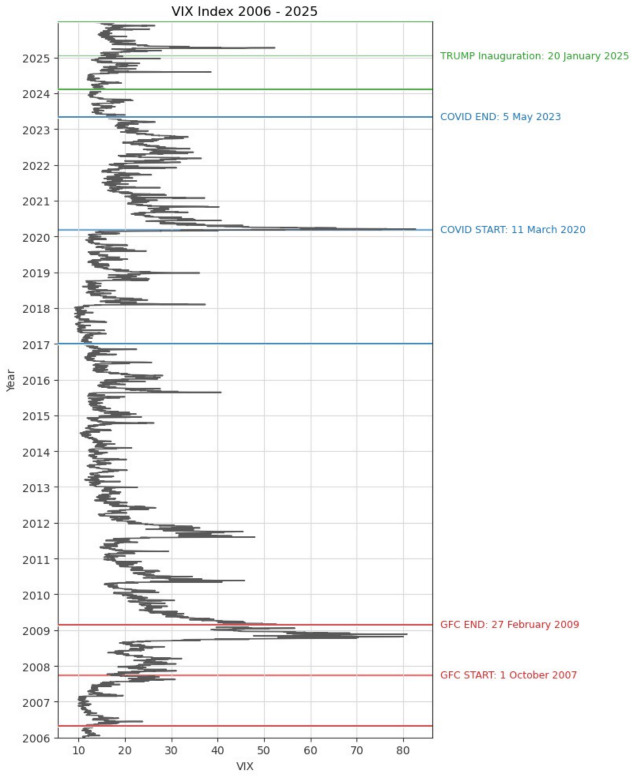
Daily VIX Index levels. Important dates for three analyzed periods are highlighted.

The subsequent Figure 2 visualizes the three aforementioned analyzed periods with important dates marked in red (the GFC), in blue (the COVID-19 pandemic), and in green (Donald Trump Inauguration).

Table 1 includes the summarized statistics for the daily VIX Index levels within the whole sample and the three investigated periods. In fact, based on Equations (1) and (2), the VIX Index level is measured in percent [3]. In general, both the median values (25.06% and 22.79%) and the mean values (32.14% and 24.55%) were especially high within the GFC and the COVID-19 pandemic periods, respectively. The standard deviation value was visibly above normal during the GFC period (15.20%). The maximal VIX Index value for the whole sample was equal to that for the COVID-19 pandemic period (82.69%). The levels of basic statistics within the period after the Donald Trump Inauguration were similar to those for the whole sample period.

### 4.2. Comparative Analysis of Symbol-Sequence Histograms

This section documents and discusses our empirical results concerning the symbol sequences, symbol-sequence histograms and related statistics. Moreover, the dynamics and the structure of patterns in the VIX data are illustrated by the charts of the related histograms.

**Figure 3:**
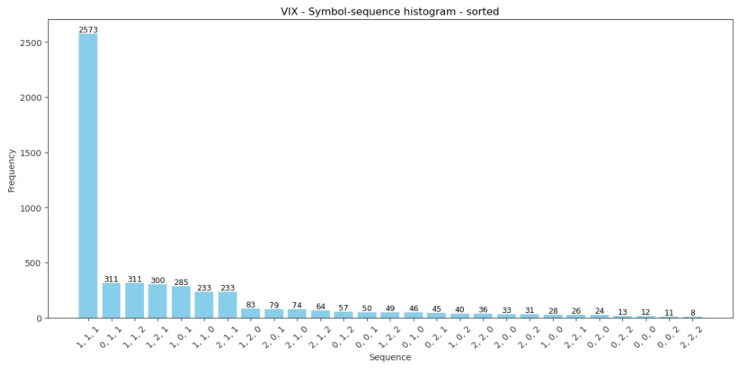
The sorted symbol-sequence histogram for the VIX Index within the whole sample period from January 2006 to December 2025.

Table 2 contains important information. First, the codes of all possible sequences (words) for the ternary alphabet A={0,1,2} and k = 3 are defined. As mentioned in Section 3.1, the number of all possible symbol sequences is equal to $n^{k}=27$. The order of sequences is based on the sorted symbol-sequence histogram for the VIX Index within the whole sample period from January 2006 to December 2025 (see Figure 3). However, the order of sequences and the assigned codes could be different and chosen arbitrarily (see, e.g., [4,8,25]).

**Figure 4:**
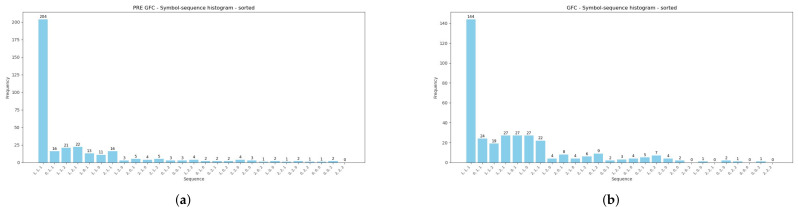
Symbol-sequence histograms based on Table 2. (**a**) The VIX symbol-sequence histogram within the pre-GFC period. (**b**) The VIX symbol-sequence histogram within the GFC period.

**Figure 5:**
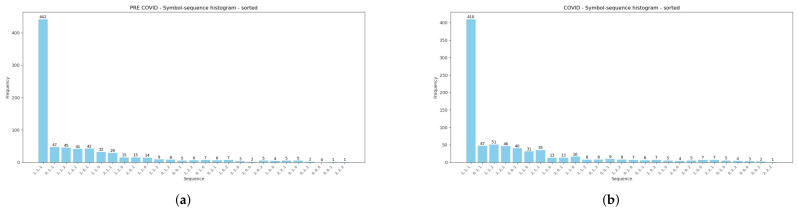
Symbol-sequence histograms based on Table 2. (**a**) The VIX symbol-sequence histogram within the pre-COVID-19 period. (**b**) The VIX symbol-sequence histogram within the COVID-19 period.

**Figure 6:**
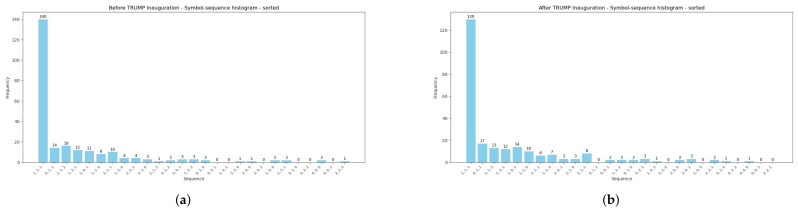
Symbol-sequence histograms based on Table 2. (**a**) The VIX symbol-sequence histogram within the period before the 2025 Donald Trump Presidential Inauguration. (**b**) The VIX symbol-sequence histogram within the period after the 2025 Donald Trump Presidential Inauguration.

Moreover, Table 2 reports summarized comparative results of encoded sequences within the pairs of sub-periods: (1) the pre-GFC and GFC, (2) the pre-COVID-19 and COVID-19 pandemic, and (3) before and after the 2025 U.S. Presidential Inauguration. Further, the Manhattan (Equation (5)) and Euclidean (Equation (6)) distances between pairs of histograms during the three analyzed periods are presented. Both measures confirm that the symbol-sequence histograms substantially differ between sub-periods, and this observation is visualized in the subsequent Figure 4, Figure 5 and Figure 6.

Figure 4, Figure 5 and Figure 6 are strictly connected with Table 2, and they illustrate symbol-sequence histograms for the pairs of sub-periods. As one can observe, all figures document a different level of regularity in the VIX Index daily logarithmic changes within the analyzed periods. Specifically, the homogeneous evidence is that regularity in the VIX Index changes decreased within each period affected by a crucial event compared to the corresponding pre-event period. Both Table 2 and Figure 4, Figure 5 and Figure 6 report the numbers of the most frequently observed sequence No. 1 (1, 1, 1). This sequence means that three successive daily logarithmic changes in the VIX level are not extremely low or high but lie between thresholds (see Definition 1). In summary, the empirical findings confirm that the (1, 1, 1) sequence dominates decidedly, and this evidence is consistent with the literature concerning the informational content of various financial time series [4,8,24,25].

### 4.3. Informational Content of the VIX Index

In this section, the comparative empirical findings of the informational content measured by normalized Shannon entropy (Definition 3) are presented and discussed. Changes in the normalized Shannon entropy values for the pre-event and event periods are calculated to assess whether entropy of the VIX Index decreased/increased during the analyzed crucial event periods.

Table 3 reports the summarized results of normalized Shannon entropy during all investigated sub-periods. The evidence is unambiguous and strictly connected with the empirical findings illustrated by the symbol-sequence histograms (Figure 4, Figure 5 and Figure 6). One can conclude that normalized Shannon entropy increased, while regularity in the VIX Index changes substantially decreased during each period affected by a crucial event compared to the corresponding pre-event period. The evidence is especially clear for the GFC and pre-GFC periods (the rise in entropy equal to 0.147). In general, higher values of entropy are related to randomness in the evolution of financial time series.

**Figure 7:**
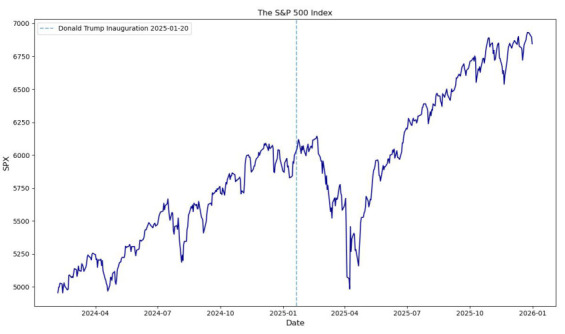
The S&P 500 Index values within the period from 7 February 2024 to 31 December 2025 (two combined periods before and after the 2025 U.S. Presidential Inauguration).

However, in comparison to the literature, the empirical findings for other financial instruments are mainly opposite. This evidence is not surprising since the VIX Index significantly differs from other indices and financial instruments. For instance, the following Figure 7 illustrates the S&P 500 Index values during the period from 7 February 2024 to 31 December 2025. The dotted vertical line shows the date of the Donald Trump Presidential Inauguration. One can observe a significant decrease in the S&P 500 Index values after the Presidential Inauguration, with the lowest value equal to 4982.77 on 8 April 2025.

In contrast, as reported in Figure 1 and Figure 2, Section 4, the VIX Index acts as a “fear index”, and high levels of VIX are coincident with high degrees of market turmoil.

The existing literature documents that different entropy-based indicators and procedures are frequently employed in assessing financial time series, especially in the context of the Efficient Market Hypothesis (EMH) [36]. The usefulness of the entropy concept in financial analyses is strictly connected with the fact that entropy is addressing system regularity and predictability, with higher entropy associated with more randomness. Most of the research relates to the weak form of market informational efficiency, since the used information set usually includes only the history of data. It is worth emphasizing that much of the theoretical literature in finance is based on market efficiency arguments that imply unpredictability in returns. However, the topic is still debatable, since the empirical evidence is rather mixed, and the findings of market informational efficiency/inefficiency are not homogeneous, especially in the context of the existence of various turbulent periods that frequently occur worldwide [7,8,24,25,37,38,39,40,41,42,43,44,45,46,47,48,49,50,51,52]. These findings are important for academics and practitioners as they support (or do not support) the hypothesis that a sequential regularity in financial time series exists and even rises during turbulent periods, which implies a possibility of returns prediction. Contrary to these findings, the unique construction of the VIX Index implies that regularity in the VIX changes substantially decreases within turbulent periods compared to the corresponding pre-turbulent periods.

### 4.4. The Evolution of Normalized Shannon Entropy over Time

In this section, the evolution of normalized Shannon entropy over time is investigated. A rolling-window dynamic approach is utilized to assess the changes in the VIX Index regularity (measured by entropy) through time. A rolling window method is one where the length of the in-sample period is fixed, so that the start date and the end date successively increase by one observation [53]. In this research, the rolling-window N = 100 trading days is used (see, e.g., [39,53]).

Normalized Shannon entropy (given by Definition 3) can be treated as a certain descriptive statistic of time series. Rolling analysis of a particular time series statistic can give an indication of structural changes or instability in the moments of a time series, and it is widely used in practice [54].

**Figure 8:**
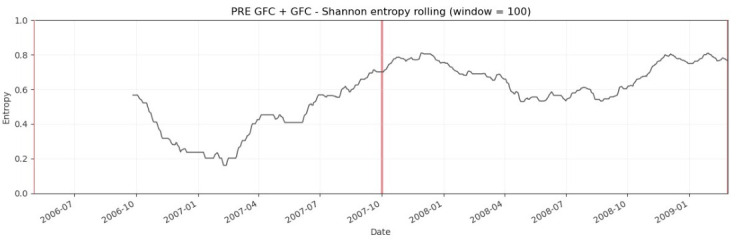
Dynamic Shannon entropy of the VIX Index within the period from 2 May 2006 to 27 February 2009 (two combined pre-GFC and GFC periods). The rolling-window N = 100 trading days. The red vertical line indicates 1 October 2007.

**Figure 9:**
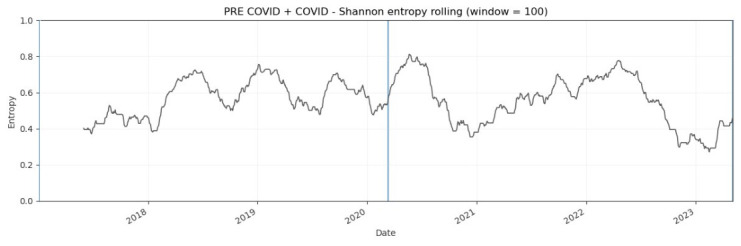
Dynamic Shannon entropy of the VIX Index within the period from 3 January 2017 to 5 May 2023 (two combined pre-COVID-19 and COVID-19 periods). The rolling-window N = 100 trading days. The blue vertical line indicates 11 March 2020.

**Figure 10:**
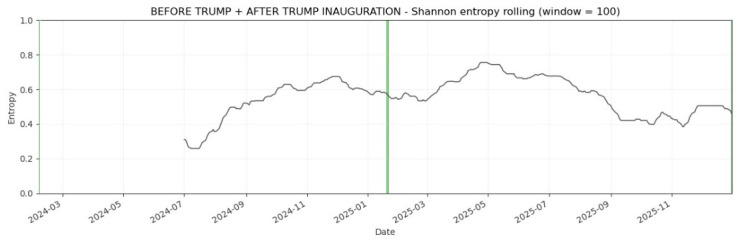
Dynamic Shannon entropy of the VIX Index within the period from 7 February 2024 to 31 December 2025 (two combined periods before and after the 2025 U.S. Presidential Inauguration). The rolling-window N = 100 trading days. The green vertical line indicates 20 January 2025.

The rolling-window empirical results usually are plotted. Figure 8, Figure 9 and Figure 10 plot the evolution of normalized Shannon entropy within three pairs of two combined pre-turbulent and turbulent periods, respectively. The vertical lines are in accord with Figure 2. As emphasized in the Introduction, the VIX Index is called the “investor fear gauge”, because the VIX level tends to spike during periods of market turmoil and various extreme events, and this feature determines the look of the charts presented in this section. Usually, the increase in the “fear” level is coupled with the entropy increase (see Table 3).

Figure 8 shows the dynamic normalized Shannon entropy of the VIX Index during the period from 2 May 2006 to 27 February 2009 (two combined pre-GFC and GFC periods). As mentioned in Section 4, the GFC timeline in the U.S. was determined by the following four major events: (1) the increase in subprime delinquency rates in the spring of 2007, (2) the liquidity and credit crunch in late 2007, (3) the liquidation of Bear Stearns in March 2008, and (4) the bankruptcy of Lehman Brothers in September 2008. The entropy estimation implemented in the rolling-window scheme reveals a consistent growth in the entropy value and confirms the influence of the aforementioned events on the VIX Index regularity.

Figure 9 illustrates the dynamic normalized Shannon entropy of the VIX Index during the period from 3 January 2017 to 5 May 2023 (two combined pre-COVID-19 and COVID-19 periods). This figure is quite interesting and worth a comment, because it documents almost a cyclical fluctuation of entropy implemented in the rolling-window scheme, specifically within the pre-COVID-19 period. This evidence is probably connected with the definition of the VIX Index, which is commonly perceived as the “fear index” [3]. There is considerable variation in the rolling estimates of daily normalized Shannon entropy values.

Figure 10 presents the dynamic normalized Shannon entropy of the VIX Index during the period from 7 February 2024 to 31 December 2025 (two combined periods before and after the 2025 U.S. Presidential Inauguration). The main evidence is that the patterns in normalized Shannon entropy calculated in the rolling-window scheme are less noticeable compared to Figure 8 and Figure 9, but the entropy increase after the Donald Trump’s Inauguration is in accord with the results reported in Table 3.

## 5. Conclusions

As mentioned in the Introduction, the contribution of this study is twofold. The main methodological contribution is the utilization of a new procedure (based on normalized Shannon entropy) to assess the informational content of the VIX Index. Specifically, the rolling-window dynamic approach is used to explore the evolution of normalized Shannon entropy over time. The main empirical contribution of our research is the thorough investigation of the influence of several event periods on the informational content of the VIX Index changes. The long time period (twenty years from 2006 to 2025), including three important periods of the twenty-first century, is analyzed. All major findings are illustrated with symbol-sequence histograms and other charts.

The empirical results presented in Section 4.3 confirm that the normalized Shannon entropy increased, since regularity in the VIX Index changes substantially decreased within each period affected by a crucial event compared to the corresponding pre-event period. However, the findings of the robustness analyses reported in Appendix A indicate that the entropy values appear to be quite sensitive to the choice of the encoding procedure. Importantly, this evidence is known and consistent with the literature [4,8,9].

In the light of the existing literature, the different indicators react more or less to periods of crisis and turmoil. However, historically, the indications of the VIX Index changes were rather clear and homogeneous. In the past, the Asian crisis (July 1997–December 1997), the Russian crisis (August 1998–September 1998), the downtrend on the main stock markets ( September 2000–September 2002), and the terrorist attacks of 11 September 2001 were coincident with corresponding visible peaks in the VIX Index level [11].

Despite some limitations of our research, we believe that the novel empirical findings reported in this paper might be interesting for both academics and investors, because the information concerning degree of time series regularity measured by entropy is strictly connected with possibility of time series prediction. Specifically, the entropy-based technique presented in our paper might be incorporated into an automatic system supporting investment decisions and strategies, for instance, as a market sentiment indicator in technical analysis [50].

A well-known good practice in each research paper is to appoint a promising avenue for future investigation. Recently, the next extreme event took place. On 28 February 2026, the Iran War was initiated by the United States and Israel. The conflict centered on Iran has upended the dynamics of the Middle East. Obviously, investors fear that a drawn-out conflict in the Middle East will hurt the global economy. In general, oil prices jumped, while equities and government bonds fell across the board as continued hostilities stoked fears of an energy-driven inflation shock that could force central banks to raise interest rate [55]. In our opinion, this crucial event has caused the great turmoil worldwide, and it certainly will be well-founded to analyze this turbulent period in the future research, specifically in the context of the VIX “fear index” performance.

## Figures and Tables

**Table 1:** Basic statistics for daily VIX Index levels within the whole sample and three analyzed periods.

Period	Median	Mean	Std. Dev.	Min	Max
Whole sample 1 January 2006–31 December 2025	17.08	19.45	8.72	9.14	82.69
GFC period 1 October 2007–27 February 2009	25.06	32.14	15.20	16.12	80.86
COVID period 12 March 2020–5 May 2023	22.79	24.55	8.15	15.01	82.69
After Trump Inauguration 21 January 2025–31 December 2025	17.21	19.03	5.37	13.47	52.33

**Table 2:** The summarized comparative results of encoded sequences for the VIX Index within the pairs of periods: (1) the pre-GFC and GFC, (2) the pre-COVID-19 and COVID-19 pandemic, and (3) before and after the 2025 U.S. Presidential Inauguration.

		The Number of Sequences					
		Global Financial Crisis		COVID-19 Pandemic		Presidential Inauguration	
Code	Sequence	Pre-GFC	GFC	Pre-COVID	COVID	Before	After
1	(1, 1, 1)	204/353	144/353	442/798	410/798	140/242	130/242
2	(0, 1, 1)	16/353	24/353	47/798	47/798	14/242	17/242
3	(1, 1, 2)	21/353	19/353	45/798	51/798	16/242	13/242
4	(1, 2, 1)	22/353	27/353	41/798	46/798	12/242	12/242
5	(1, 0, 1)	13/353	27/353	42/798	40/798	11/242	14/242
6	(1, 1, 0)	11/353	27/353	32/798	31/798	8/242	10/242
7	(2, 1, 1)	16/353	22/353	29/798	35/798	10/242	6/242
8	(1, 2, 0)	3/353	4/353	15/798	13/798	4/242	7/242
9	(2, 0, 1)	5/353	8/353	15/798	13/798	4/242	3/242
10	(2, 1, 0)	4/353	4/353	14/798	16/798	3/242	3/242
11	(2, 1, 2)	5/353	6/353	9/798	8/798	1/242	8/242
12	(0, 1, 2)	3/353	9/353	8/798	8/798	2/242	0/242
13	(0, 0, 1)	3/353	2/353	5/798	9/798	3/242	2/242
14	(1, 2, 2)	4/353	3/353	6/798	8/798	3/242	2/242
15	(0, 1, 0)	2/353	4/353	7/798	7/798	2/242	2/242
16	(0, 2, 1)	2/353	5/353	6/798	6/798	0/242	3/242
17	(1, 0, 2)	2/353	7/353	7/798	7/798	0/242	1/242
18	(2, 2, 0)	4/353	4/353	3/798	5/798	1/242	0/242
19	(2, 0, 0)	3/353	2/353	2/798	4/798	1/242	2/242
20	(2, 0, 2)	1/353	0/353	5/798	5/798	0/242	3/242
21	(1, 0, 0)	2/353	1/353	4/798	7/798	2/242	0/242
22	(2, 2, 1)	1/353	0/353	5/798	7/798	2/242	2/242
23	(0, 2, 0)	2/353	2/353	5/798	5/798	0/242	1/242
24	(0, 2, 2)	1/353	1/353	2/798	4/798	0/242	0/242
25	(0, 0, 0)	1/353	0/353	0/798	3/798	2/242	1/242
26	(0, 0, 2)	2/353	1/353	1/798	2/798	0/242	0/242
27	(2, 2, 2)	0/353	0/353	1/798	1/798	1/242	0/242
Manhattan distance		140		80		54	
Euclidean distance		65.38		34.55		15.49	

The numbers of all sequences within sub-periods are equal to (1) 353 (the GFC), (2) 798 (the COVID-19 pandemic), and (3) 242 (the 2025 U.S. Presidential Inauguration).

**Table 3:** The normalized Shannon entropy (Definition 3).

The Normalized Shannon Entropy	
Pre-GFC period	0.566
GFC period	0.713
Change in entropy	Δ=0.147↑
Pre-COVID-19 period	0.586
COVID-19 period	0.625
Change in entropy	Δ=0.039↑
Before Donald Trump’s Inauguration	0.586
After Donald Trump Inauguration	0.629
Change in entropy	Δ=0.043↑

The up arrow shows an entropy increase.

## Data Availability

The free-of-charge data come from the Federal Reserve Bank of St. Louis (FRED) database (https://fred.stlouisfed.org/series/VIXCLS, accessed on 3 January 2026).

## Abbreviations

The following abbreviations are used in this manuscript:

VIX	The CBOE Volatility Index
CBOE	Chicago Board Options Exchange
SPX	The S&P 500 Index
STSA	Symbolic Time-Series Analysis
GFC	Global Financial Crisis (2007–2009)
COVID-19	COVID-19 Pandemic
EMH	Efficient Market Hypothesis

## Appendix A. Alternative Ternary Discretization Procedures: Robustness Analyses

To perform robustness analyses, three alternative ternary discretization procedures are used. Definitions A1 and A2 are the modifications of Definition 1 (we would like to thank an anonymous referee for this suggestion), while Definition A3 is the variant of the STSA method with the 5% and 95% sample quantiles as the thresholds.

**Definition** **A1.**
*A sequence $\left\{s_{t}\right\}$ of symbols is defined according to the following:*

(A1)
$$s_{t}=\left\{\begin{array}{cc} 0 & if\;r_{t}\leq \left(\bar{r}-0.5\cdot \sigma \right)\\ 1 & if\;\left(\bar{r}-0.5\cdot \sigma \right)<r_{t}\leq \left(\bar{r}+0.5\cdot \sigma \right)\\ 2 & if\;r_{t}>\left(\bar{r}+0.5\cdot \sigma \right) \end{array}\right.$$

*where $\bar{r}$ is the mean of logarithmic changes time series, σ is the standard deviation of logarithmic changes, and $\left(\bar{r}-0.5\cdot \sigma \right)$ and $\left(\bar{r}+0.5\cdot \sigma \right)$ are the thresholds of the time series.*

**Definition** **A2.**
*A sequence $\left\{s_{t}\right\}$ of symbols is defined according to the following:*

(A2)
$$s_{t}=\left\{\begin{array}{cc} 0 & if\;r_{t}\leq \left(\bar{r}-1.5\cdot \sigma \right)\\ 1 & if\;\left(\bar{r}-1.5\cdot \sigma \right)<r_{t}\leq \left(\bar{r}+1.5\cdot \sigma \right)\\ 2 & if\;r_{t}>\left(\bar{r}+1.5\cdot \sigma \right) \end{array}\right.$$

*where $\bar{r}$ is the mean of logarithmic changes time series, σ is the standard deviation of logarithmic changes, and $\left(\bar{r}-1.5\cdot \sigma \right)$ and $\left(\bar{r}+1.5\cdot \sigma \right)$ are the thresholds of the time series.*

**Definition** **A3.**
*A sequence $\left\{s_{t}\right\}$ of symbols is defined according to*

(A3)
$$s_{t}=\left\{\begin{array}{cc} 0 & if\;x_{t}\leq \theta_{1}\\ 1 & if\;\theta_{1}<x_{t}\leq \theta_{2}\\ 2 & if\;x_{t}>\theta_{2} \end{array}\right.$$

*where $\theta_{1}$ is the 5% sample quantile, and $\theta_{2}$ is the 95% sample quantile [4,8,21,22].*

Table A1 reports the summarized findings of the normalized Shannon entropy during all investigated sub-periods (based on Definitions A1–A3).

One can observe that only the results obtained using Definition A1 are unambiguous and consistent with the results reported in Table 3 (based on Definition 1). The normalized Shannon entropy increased during each period affected by a crucial event compared to the corresponding pre-event period.

For thresholds defined by Definition A1, entropy attains its highest values. Under this specification, the thresholds are relatively narrow, which causes a larger proportion of observations to be assigned to the extreme classes, 0 and 2. As a result, the distribution of sequences becomes more diverse. In contrast, for thresholds defined Definition A2, entropy decreases markedly. In this case, the thresholds are substantially wider; so, most observations are classified into the middle class, 1. Consequently, the sequence (1, 1, 1) begins to dominate, leading to a reduction in sequence variability. A similar effect is observed for the quantile-based thresholds (Definition A3). As with the wider mean-based thresholds, there is a pronounced dominance of the middle class, and the resulting entropy values are comparable to those obtained for Definition A2 specification. Overall, the entropy values appear to be quite sensitive to the choice of coding thresholds; however, the direction of change remains consistent. Narrower thresholds lead to higher sequence diversity and higher entropy, whereas wider thresholds result in stronger concentration around the symbol 1 and, consequently, lower entropy. Importantly, this evidence is known and consistent with the existing literature (see, e.g., [4,8,9]).

**Table A1:** The normalized Shannon entropy based on Definitions A1–A3.

The Normalized Shannon Entropy			
	Definition A1	Definition A2	Definition A3
Pre-GFC period	0.920	0.442	0.427
GFC period	0.959	0.442	0.379
Change in entropy	Δ=0.039↑	Δ=0.000	Δ=−0.048↓
Pre-COVID-19 period	0.919	0.354	0.361
COVID-19 period	0.952	0.352	0.361
Change in entropy	Δ=0.033↑	Δ=−0.002↓	Δ=0.000
Before Donald Trump’s Inauguration	0.873	0.334	0.409
After Donald Trump Inauguration	0.903	0.402	0.422
Change in entropy	Δ=0.030↑	Δ=0.068↑	Δ=0.013↑

The up arrow shows an entropy increase while the down arrow shows an entropy decrease.
